# Internal and external modulation of parieto-premotor circuitry in movement disorders

**DOI:** 10.1093/braincomms/fcae339

**Published:** 2024-10-09

**Authors:** Anne Marthe Meppelink, Bauke M de Jong, Martijn Beudel

**Affiliations:** Stichting Epilepsie Instellingen Nederland (SEIN), 8025 BV Zwolle, The Netherlands; Department of Neurology, University Medical Center Groningen, University of Groningen, 9713 GZ Groningen, The Netherlands; Department of Neurology, Amsterdam University Medical Centers, Amsterdam Neuroscience, University of Amsterdam, 1105 AZ Amsterdam, The Netherlands

## Abstract

This scientific commentary refers to ‘Increased beta synchronization underlies perception-action hyperbinding in functional movement disorders’, by Pastötter *et al*. (https://doi.org/10.1093/braincomms/fcae301).

**This scientific commentary refers to ‘Increased beta synchronization underlies perception-action hyperbinding in functional movement disorders’, by Pastötter *et al*. (** https://doi.org/10.1093/braincomms/fcae301 **).**

In their recent paper in *Brain Communications*, Pastötter *et al*.^1^ have made an important contribution to the pathophysiological understanding of not only functional movement disorders but also movement disorders in general. Their key findings, namely that post-movement beta (±13–30 Hz) oscillatory synchronization is increased in functional movement disorders and that it predicts stimulus response binding, shed important light on how sensorimotor (dis) integration is established in health and disease.

In this Scientific Commentary, we will place the findings of Pastötter *et al*. in a broader context and will reflect on the potential diagnostic and therapeutic possibilities of their findings. The finding that (functional) movement disorders are caused by pathology not only in the motor but also in the sensory system is firmly embedded in the literature. Important examples of this are the benefits of the so-called *sensory trick*^2^ in dystonia patients and the positive role of sensory cueing and perceived *optic flow* on freezing of gait in Parkinson’s disease.^3^ Furthermore, even the electrical stimulation of the sensory system, more specifically the dorsal columns of the spinal cord, has a positive effect of movement (locomotion) in Parkinson’s disease.^4^ This raises the overall hypothesis that providing the appropriate sensory information, provided either via the senses or electrically, could be used as a therapeutic strategy to improve symptoms in movement disorders. This fits within the current concept of the emergence of functional movement disorders due to disrupted agency, attention and priors about the body scheme.^5^

When providing these examples, the question raises whether there is a unifying neural mechanism that synthesizes the sensory information for successful motor control. The localization of both the differences with healthy volunteers and the correlation with stimulus response binding in specifically the supplementary motor area (SMA) is striking and fits with existing literature on motor control. More specifically, the SMA is the location where the *Bereitschaftspotential (BP),*^6^ the most well-studied neurophysiological phenomenon in functional movement disorders, has its origin. Interestingly, the activation of this area, recorded with functional magnetic resonance imaging, has been linked with the self-regulation of movements in Parkinson’s disease.^7^ Next to this, increased SMA activity has been linked to the OFF state in Parkinson’s disease. Vice versa, the reversal of this pathological activity occurred with subthalamic nucleus (STN) deep brain stimulation, recorded with magnetoencephalography, while OFF symptoms improved after deep brain stimulation.^8^ Furthermore, real-time functional magnetic resonance imaging-based neuro-feedback of the SMA resulted in a temporary improvement of Parkinson’s disease symptoms.^8^

Apart from the similarities in *spatial location* (i.e. the SMA), Parkinson’s disease and functional movement disorders also share a common pathophysiological mechanism in the *temporal domain.* The increased synchronization in the beta band that was observed by Pastötter *et al*. has also been extensively observed in Parkinson’s disease and linked to the OFF dopaminergic and OFF deep brain stimulation state.^8^ More specifically, the power of the beta activity in the STN is correlated with the severity of contralateral bradykinesia and rigidity. Interestingly, the pathological activity in the beta band is likely to be mediated via the hyperdirect pathway from the motor cortex, including the SMA, to the STN. More evidence from a pathological link between SMA and STN comes from other neuro-feedback studies based on local field potentials derived from implanted deep brain stimulation electrodes.^9^ With this method, Parkinson’s disease patients can observe their amount of STN beta activity in real time and are even able to decrease this using neuro-feedback.^9^ This provides evidence that even the pathological neural activity can be influenced by both internal and external modulations.

The cortical and subcortical beta activity typically decreases (lateralized to the contralateral side of the movement) before voluntary movement. This beta suppression, also coined event-related desynchronization, has also been observed prior to functional jerks and can be used as a diagnostic marker for distinguishing functional movement disorders from myoclonus.^6^ In addition, patients with functional movement disorders showed lower beta desynchronization prior to intended movement when compared to healthy controls.^10^ While the focus of this study was on motor preparation (before and during movement), its data suggests a similar pattern like Pastötter *et al*., of persisting beta synchronization in patients with functional movement disorders *after* motor execution.

One explanation for this is an excessive attentional focus on monitoring the current state of the limb to be moved, combined with a decrease of attention towards the goal of movement.^6^ This is reflected by a key clinical characteristic of movement patterns in patients with functional movement disorders, showing failure of explicitly controlled movement with major improvement or even full recovery when the same movement occurs in an implicit or unattended fashion.^5^ Interestingly, this dissociation between performance of implicit and explicit motor control is also seen in patients with Parkinson’s disease.

The inability to suppress beta synchronization and reduced stimulus response binding in functional movement disorders as shown by Pastötter *et al.* is in line with the theory of a hyperattentive neural state where the patient is ‘stuck’ in the current motor state. However, the overall neural dynamics of functional movement disorders, with diverse motor phenotypes, are more difficult to explain and may necessitate a broader network approach.

With regard to voluntary action selection, the SMA is known to play an important role concerning internally driven action, while more posterior regions are involved in sensorimotor integration contributing to the explicit volition to act. Regarding the latter, tuning sensorimotor predictions and actual feedback by parietal–cerebellar computations appears to be a mechanism crucially involved in generating a sense of agency.^11^ Intra-operative studies have shown that electrical stimulation of the SMA can lead to overt motor activation without conscious perception (i.e. subjects denied that they moved). This lack of *agency* of executed movement is also one of the key features of functional movement disorders. While stimulation of the SMA led to movement without awareness, activation of the (right) inferior parietal regions evoked a sense of motor intention and even the *conviction* of *having actually moved* with greater stimulation output.^12^

A possible functional disconnection within the parieto-premotor network may underlie motor impairment in functional movement disorders and possibly also in other movement disorders, which may be reflected by changes in beta synchronization.
